# Eubulus Williams

**Published:** 1905-03

**Authors:** 


### EUBULUS WILLIAMS, M.D., M.R.C.S.

It is with sincere regret we notice the death of Dr. E. Williams on January 9th, in his 74th year. Born at Williton, Somerset, he received his medical education at Guy's Hospital. After qualifying he was appointed house-surgeon to Reading Hospital in 1854, and in 1855 was appointed the first medical superintendent to the Dundee Royal Infirmary. In those days typhus fever was endemic in Dundee, and his experience in that disease, which is nowadays unknown to most practitioners, was considerable—and personal, for he nearly succumbed to an attack contracted during his work in the infirmary. After nearly three years' residence, he spent some time studying in the hospitals of Paris and Vienna. He settled in practice in Bristol in 1858, soon afterwards marrying the daughter of the late Patrick Watson, of Dundee. Dr. Williams was associated with Dr. Mortimer Granville and the late Dr. Henderson at the dispensary in St. James' Square for "diseases of women and children." This work prospered, and ultimately led to the starting of the Children's Hospital on St. Michael's Hill, when Dr. Williams became a member of the surgical staff. After a time he resigned, owing to pressure of engagements, but was re-elected surgeon in 1863. In 1867, when his practice was rapidly increasing, he was induced by his friend, Dr. Gibbs Blake, of Birmingham, to investigate and test the principles of homœopathy, and in consequence of convictions which we have good reason to know were sincere he resigned his appointment at the hospital which he so dearly cherished. He continued to practice up to within a short time of his death.

For thirty-four years he was physician to Müller's Orphanage, in which great philanthropic institution he ever took the keenest interest. We cordially endorse the remarks of the Bristol press that "it will be judged from his life-history that he was a man of independent thought; he had a kindly disposition and wide sympathies"; and though we cannot pretend to sympathise with the therapeutical views he held and practised, we can admire the honesty and sincerity of purpose which won for him not only the confidence of his patients, but the warm regard of many members of the medical profession of Bristol, and none the less for his remaining faithful to his convictions to the end.

